# Quantitative X-ray tomographic analysis reveals calcium precipitation in cataractogenesis

**DOI:** 10.1038/s41598-021-96867-7

**Published:** 2021-08-31

**Authors:** Yuxing Li, Dilworth Y. Parkinson, Jun Feng, Chun-hong Xia, Xiaohua Gong

**Affiliations:** 1 Vision Science Program and School of Optometry, University of California, Berkeley, 693 Minor Hall, Berkeley, CA 94720-2020 USA; 2 Tsinghua-Berkeley Shenzhen Institute (TBSI), UC Berkeley, Berkeley, CA USA; 3 Advanced Light Source Division, Lawrence Berkeley National Laboratory, Berkeley, CA USA

**Keywords:** Biophysics, Diseases, Eye diseases

## Abstract

Cataracts, named for pathological light scattering in the lens, are known to be associated with increased large protein aggregates, disrupted protein phase separation, and/or osmotic imbalances in lens cells. We have applied synchrotron phase contrast X-ray micro-computed tomography to directly examine an age-related nuclear cataract model in Cx46 knockout (Cx46KO) mice. High-resolution 3D X-ray tomographic images reveal amorphous spots and strip-like dense matter precipitates in lens cores of all examined Cx46KO mice at different ages. The precipitates are predominantly accumulated in the anterior suture regions of lens cores, and they become longer and dense as mice age. Alizarin red staining data confirms the presence of calcium precipitates in lens cores of all Cx46KO mice. This study indicates that the spatial and temporal calcium precipitation is an age-related event associated with age-related nuclear cataract formation in Cx46KO mice, and further suggests that the loss of Cx46 promotes calcium precipitates in the lens core, which is a new mechanism that likely contributes to the pathological light scattering in this age-related cataract model.

## Introduction

Cataracts, named for lens opacity, are clinically examined by a light imaging-based slit-lamp microscopy and are evaluated for the degree of opacities according to Lens Opacities Classification System III (LOCS III)^1^. Cataract etiology is related to gene mutations, aging, radiation, metabolic diseases such as diabetes, and many other risk factors. Extensive biochemical, molecular and cellular biological, and biophysical studies in vitro indicate that the lens light scattering is associated with increased abnormal lens protein aggregates, disrupted osmotic balances of lens cells, and/or altered phase separation of crystallin proteins. However, there are limited studies to directly address the actual components that cause abnormal light scattering of cataracts in live lenses, animals, or human subjects. Such knowledge is critical for further understanding the molecular and cellular mechanisms of cataract formation and lens pathology in vivo.

The lens is an avascular organ composed of a monolayer of epithelium covering the anterior side of bulk of elongated fiber cells, and it is wrapped by a basement membrane called lens capsule. The lens grows throughout life; equatorial surface epithelial cells differentiate into elongated fiber cells that precisely overlay onto previous generations of fiber cells, and interior fiber cells undergo cell maturation to eliminate intracellular organelles including nucleus, mitochondrion, endoplasmic reticulum and Golgi apparatus for lens transparency^2,3^. Lens homeostasis has been suggested to rely on an internal circulation system, in which sodium ionic currents enter from anterior and posterior regions into interior lens fibers, and they then pass through intercellular gap junction channels to reach lens equatorial surface fibers and epithelial cells to be transported out of the lens by sodium–potassium ATPase pumps^4^. Disruption of either Cx46 or Cx50, encoded by Gja3 or Gja8 genes, abolishes intercellular gap junction communication to impair fiber-to-fiber coupling, which disrupts lens current circulation and homeostasis, leading to different types of cataracts and/or smaller lenses^5–9^. Mutations of Cx46 and Cx50 have been reported to be associated with different types of cataracts in humans^10–13^.

Cx46 knockout (Cx46KO) mice develop a postnatal age-related nuclear cataract and abnormal light scattering in the lens core^14–16^. The severe nuclear cataract of Cx46KO lenses is associated with increased water-insoluble crystallin proteins and increased protein degradation in the lens core. Moreover, mouse strain backgrounds also significantly affect the cataract severity. Severe nuclear cataracts appear in Cx46KO lenses of 129 (both 129Svjae and 129SvJ) strains while mild nuclear cataracts develop in the C57BL/6J (B6) strain^17^.

Calcium elevation and the presence of calpain3 protease have been reported to be directly associated with severe nuclear cataracts in Cx46KO lenses^14,16,18^. Thus, light scattering of severe nuclear cataracts is suggested to be associated with the calpain3-mediated cleavage of crystallin proteins to trigger abnormal protein aggregates that directly cause light scattering in the Cx46KO lens cores. However, the biophysical and biochemical nature of the components that cause cataractous light scattering in vivo has never been directly examined in Cx46KO lenses due to the lack of appropriate technology.

Without the injection of contrast agents, synchrotron X-rays with an energy of 20 keV provide a novel biomedical imaging approach for biological samples^19–21^. High-resolution X-ray CT phase-contrast image analysis has previously been reported to examine enucleated and intracranial rabbit eyes^22^ and internal structures of formalin-fixed monkey eyes^23^. Here we report a study of mouse Cx46KO live lenses using three-dimensional (3D) morphometric analysis of micro computed-tomography (μCT) images^24,25^. High-resolution 3D X-ray phase-contrast images reveal age-related increases in the amount and distribution of calcium precipitates, matching age-related nuclear cataracts in Cx46KO lens cores. This work sheds light on new mechanistic information about how accumulated calcium precipitates contribute to age-related nuclear cataracts in Cx46KO mice.

## Results

### X-ray electron-dense precipitates are highly accumulated in Cx46KO nuclear cataract

**Figure 1:**
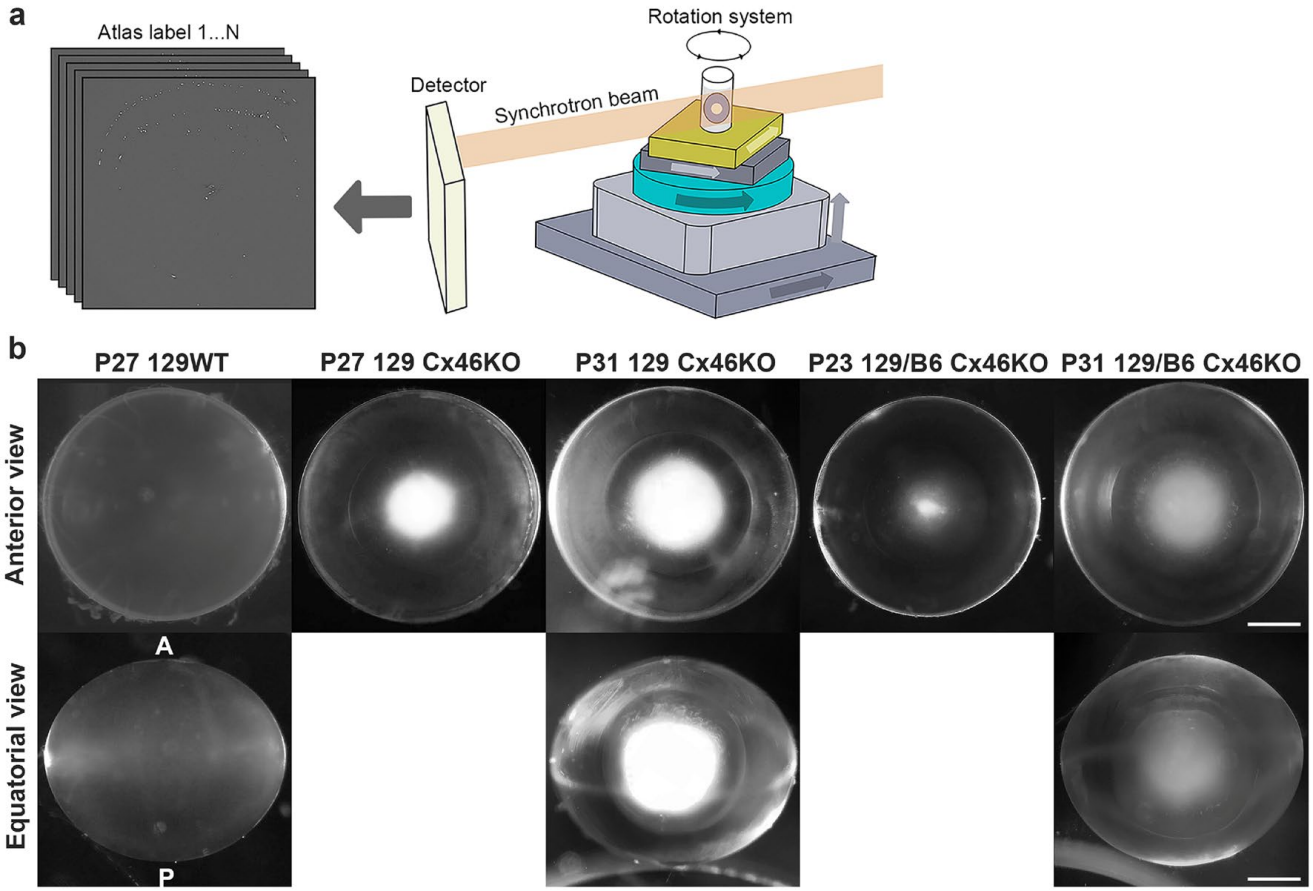
Micro computed-tomographic (μCT) image scanning of fresh mouse lenses. (**a**) A μCT scanner includes a rotation system for placing the lens sample to be examined by the synchrotron beam, and a detector for collecting phase-contrast images. (**b**) Lens images of 129 wild-type (129WT), 129 Cx46KO, and 129/B6 Cx46KO mice at postnatal days 23–31 under the dissecting microscope. Anterior lens views and equatorial views of some lenses were shown; the anterior (A) and posterior (P) poles were indicated on the left image of the equatorial images. Scale bar: 0.5 mm.

**Figure 2:**
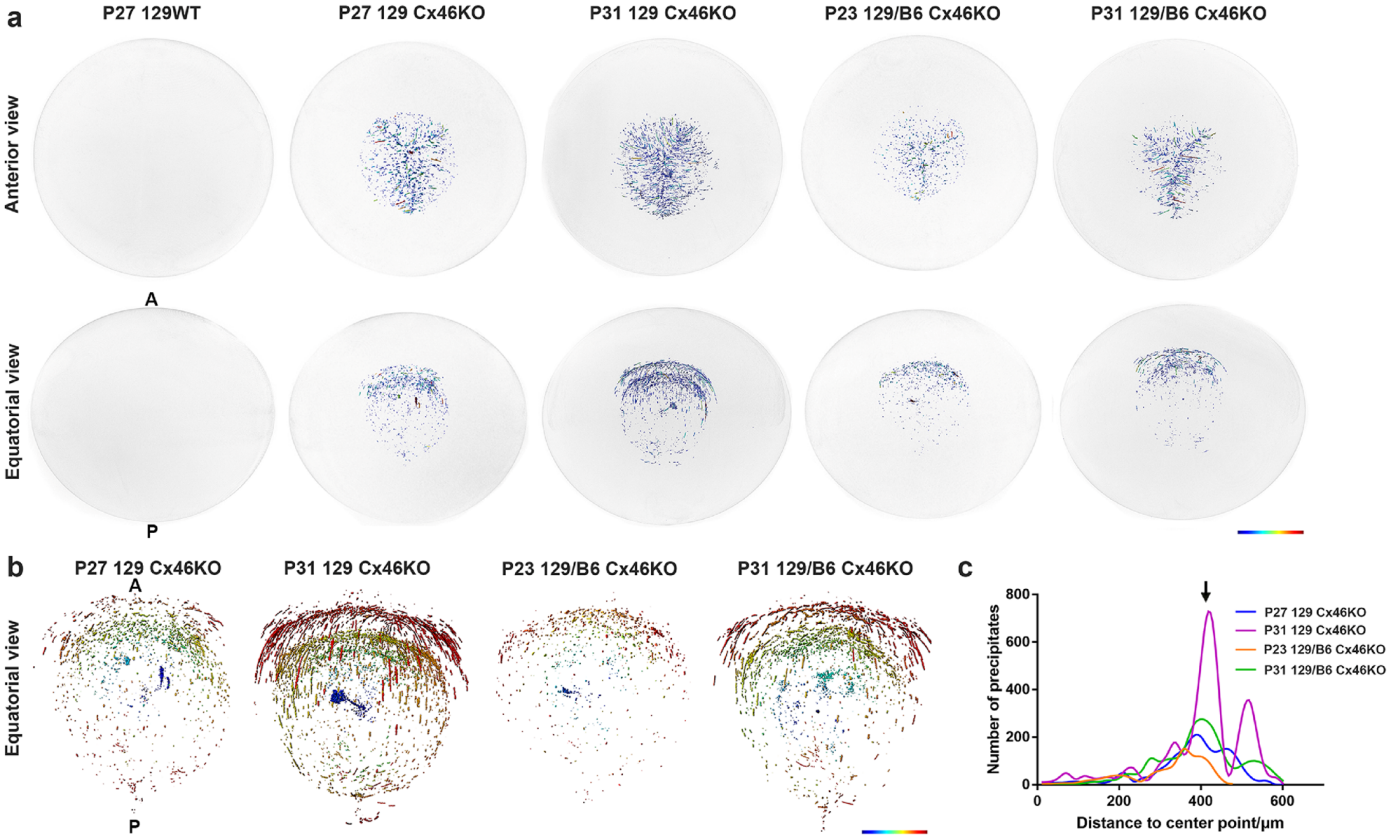
Lens μCT images show the distribution of electron-dense precipitates in Cx46KO lenses. (**a**) 3D visualization of reconstructed μCT images of 129 wild-type, 129 Cx46KO, and 129/B6 Cx46KO lenses, the same lenses shown in Fig. 1. Each individual lens was used for both anterior and equatorial image analysis. Electron-dense precipitates, shown as dark blue dots, were only detected in the cataractous regions of all Cx46KO lenses but not in the wild-type control lens. For the equatorial images, lens anterior (A) and posterior (P) poles were indicated on the left image. (**b**) Enlarged equatorial images were false colored depicting the aggregate distance of individually colored precipitates to the central point of two 129 Cx46KO lenses (P27 and P31) and two 129/B6 Cx46KO lenses (P23 and P31), magenta/red color representing further away from the center point and blue/green representing close to the center point. (**c**) Quantitative distribution graph shows the total number of individual precipitates versus their distances to the lens center point for the images in (**b**). The major peak of precipitates (indicated by an arrow) is located at around 400 µm from the lens center point. This peak is higher in P31 129 Cx46KO than in P27 129 Cx46KO, and it is also higher in P31 129/B6 Cx46KO than in the P23 129/B6 Cx46KO.

Phase contrast μCT with Beamline 8.3.2 at the Advanced Light Source was used for acquiring the X-ray tomography of fresh lenses from 129 wild-type (WT), 129 Cx46KO and 129/B6 Cx46KO mice (Fig. 1a). As expected, 129WT lenses were clear and Cx46KO lenses showed age-related nuclear cataracts. The 129 Cx46KO lenses at postnatal day 31 (P31) had more severe nuclear cataract than P27 129 Cx46KO lenses, and P31 129/B6 Cx46KO lenses also showed more severe nuclear cataract than P23 129/B6 Cx46KO lenses (Fig. 1b). These fresh lenses were subjected to 3D μCT imaging analysis. We performed 3D μCT imaging on a total of eight lenses: two wild-type lenses at P21 and P27, and six Cx46KO lenses, including three 129 Cx46KO lenses (one at P27 and two at P31) and three 129/B6 Cx46KO lenses (one at P23 and two at P31). All Cx46KO lenses had electron-dense matters named for precipitates in the lens cores while the 129WT lens was transparent without any precipitates. Reconstructed μCT images, from both anterior view (along the visual axis) and equatorial view (from the lens side, indicated with A for anterior and P for posterior poles) of 3D imaging data of individual Cx46KO lenses, showed that these electron-dense precipitates (dark blue dots) were enriched in the anterior parts of Cx46KO lens cores (Fig. 2a). P31 129 Cx46KO lens displayed more precipitates than P27 129 Cx46KO lens and P31 129/B6 Cx46KO lens showed more precipitates than P23 129/B6 Cx46KO lens. Thus, we performed detailed quantitative analysis of these precipitates in individual lenses of P27 and P31 129 Cx46KO, and P23 and P31 129/B6 Cx46KO.

High-magnified equatorial view images depicted the distribution of individually colored precipitates upon their distances to the lens central point. The close precipitates to lens central points were shown in blue/green color and the far-away precipitates were shown in magenta/red color (Fig. 2b). Precipitates of P31 129 Cx46KO and P31 129/B6 Cx46KO lenses formed two enriched shell-like layers in the anterior sides of the lens cores while precipitates of P27 129 Cx46KO and P23 129/B6 Cx46KO lenses displayed less obvious shell-like layers. A quantitative analysis of all precipitates versus their distances to the lens centers of four Cx46KO lenses revealed two peaks about 100 µm apart, one located at about 420 µm and the other located at 520 µm distances from the lens center (Fig. 2c). These two peaks correspond to two shell-like layers in the high-magnified equatorial view images of P31 Cx46KO lenses.

These results indicated that both the amount and location of these precipitates seemed to be correlated with age-related nuclear cataract of Cx46KO mice at 129 and 129/B6 strain backgrounds. Therefore, we further performed high-resolution quantitative analysis of precipitates’ geometrical parameters as well as absorption from these 129 and 129/B6 Cx46KO lenses.

### Precipitates predominantly accumulate around the anterior Y suture of Cx46KO lenses

**Figure 3:**
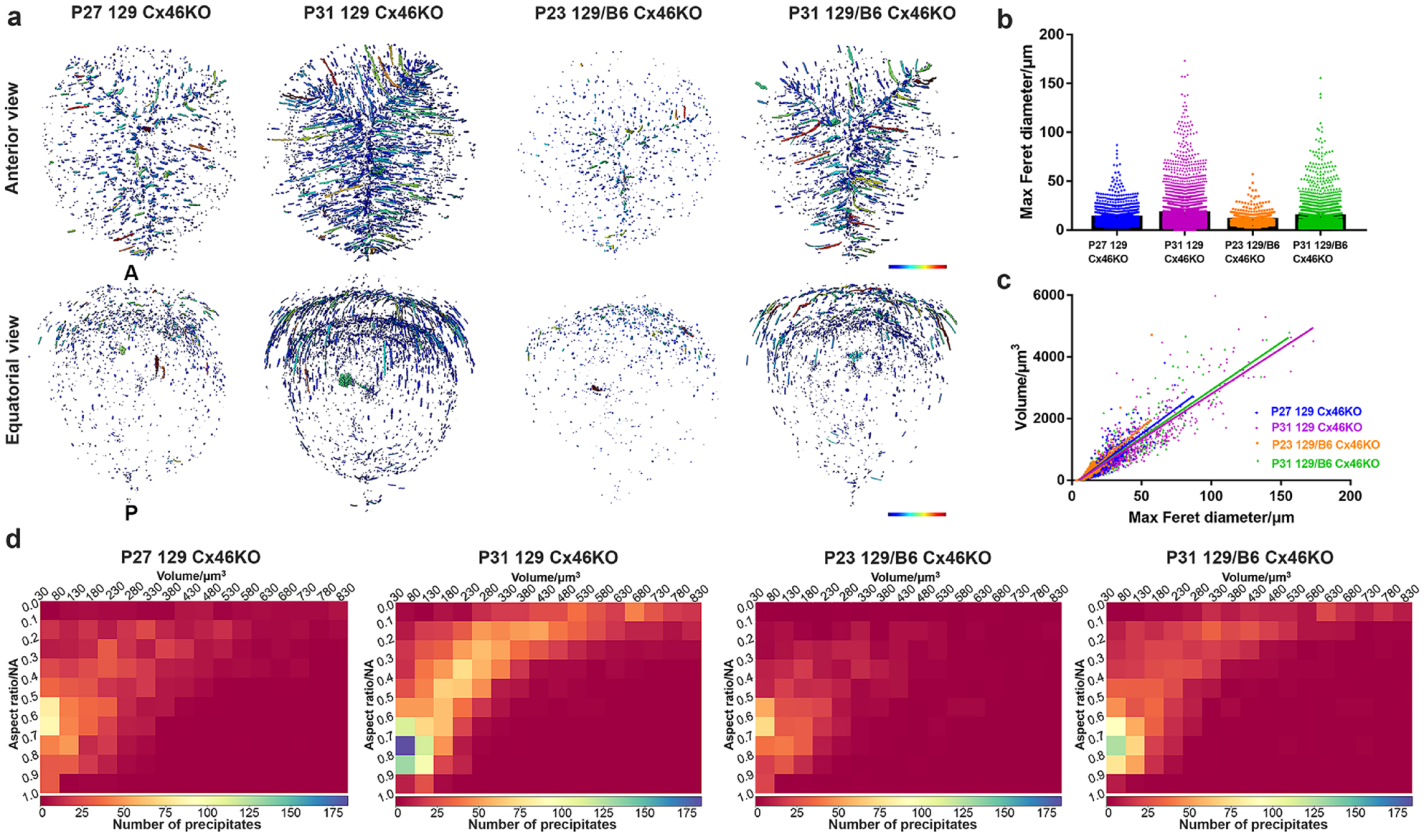
Precipitate geometrical parameter measurement of Cx46KO lenses. The same individual lenses in Fig. 2 were analyzed. (**a**) The distribution and quantification of precipitates were measured by the Max Feret diameter (length) in the cataract regions of 129 Cx46KO and 129/B6 Cx46KO lenses, scanned with the resolution of 0.65 μm/pixel. Individual precipitates, in both anterior and equatorial views, were colored based on the color bar, magenta/red for long precipitates and blue/green for short precipitates. Lens anterior (A) and posterior (P) poles were indicated on the left equatorial image. (**b**) A bar graph shows Max Feret diameter measurement of four different Cx46KO lenses. (**c**) The relationship between Max Feret diameter and volume of the precipitates in four different Cx46KO lenses. (**d**) Heat maps reveal the relationship among aspect ratio, volume, and number of precipitates in cataracts of Cx46KO lenses.

The analysis of geometrical parameter was applied to characterize these precipitates in the lens cores of all Cx46KO lenses. The distribution and quantification of precipitates, which were scanned with the resolution of 0.65 μm/pixel, were measured for the Maximum Feret diameter (MFD) that represents the longest dimension of the precipitates (Fig. 3). The Feret diameter is a measure of an object size along a specified direction and can be defined as the distance between the two parallel planes restricting the object perpendicular to that direction. All individual precipitates in both anterior and equatorial views of P27 and P31 129 Cx46KO as well as P23 and P31 129/B6 Cx46KO lenses were colored based on their MFD values with magenta/red for long precipitates and blue/green for short precipitates (Fig. 3a).

Cx46KO lenses developed age-related nuclear cataracts^16^. As expected, both the number and MFD value of precipitates in the lens core of P31 129 Cx46KO were higher than those of P27 129 Cx46KO, and the precipitate number and MFD value of P31 129/B6 Cx46KO lens core were higher than those of P23 129/B6 Cx46KO. P31 129 Cx46KO lens core had more long precipitates than P27 129 Cx46KO lens core, and P31 129/B6 Cx46KO had more long precipitates than P23 129/B6 Cx46KO lens core. Moreover, these long precipitates in P31 129 Cx46KO and P31 129/B6 Cx46KO lens core were mainly oriented and distributed along elongated fibers in the anterior Y suture regions (anterior views of Fig. 3a). The Y suture is where the ends of elongated fibers from opposite sides of the lens contact each other^26,27^. The equatorial view of P31 129 Cx46KO and P31 129/B6 Cx46KO MFD images further confirmed that the precipitates were enriched in two shell-like layers only in the anterior part, not in the posterior part of the lens core (equatorial view in Fig. 3a). The P27 129 Cx46KO MFD image showed fewer precipitates than P31 129 Cx46KO, and the P23 129/B6 Cx46KO MFD image had much fewer precipitates than P31 129/B6 Cx46KO.

A bar graph shows age-related increase of MFD precipitates in Cx46KO lenses of both 129 and 129/B6 strain backgrounds (Fig. 3b). For example, the changes of the percentages of precipitates that were below 40 µm in MFD measurement were about 99.5% and 94% for P23 and P31 129/B6 Cx46KO samples respectively, and 96% and 90% for P27 and P31 129 Cx46KO samples respectively. Only P31 Cx46KO samples had precipitates above 100 µm but below 200 µm. Except for the difference in precipitate number, there is a linear relationship between precipitate volume and MFD in all individual Cx46KO samples (Fig. 3c). The trend of MFD increase among these samples was nearly the same, indicating that the chemical nature of precipitates might be the same.

The precipitates were further quantified for the aspect ratio (AR) value, which is the ratio of precipitate size in different dimensions. AR value varies from 0 to 1 based on the precipitate shape; 0 approximates a long chain shape and 1 approximates a sphere shape. Heat maps revealed the relationship between AR value, volume and precipitate numbers in these four Cx46KO lens cores (Fig. 3d). An increase in volume and a decrease in AR value are synchronous in all Cx46KO samples, indicating that the aggregation of these precipitates preferentially extends longitudinally rather than as a whole. The P31 129 Cx46KO lens had much more long chain shape precipitates than P27 Cx46KO lens, and the P31 129/B6 Cx46KO lens contained more long chain shape precipitates than P23 129/B6 Cx46KO lens. The results match the direct observation of anterior view images (Fig. 3a). Thus, these data suggest that the longitudinal increase of precipitates is also an age-related change in Cx46KO lens core.

### Increased absorption density and precipitate numbers are correlated with cataract severity

**Figure 4:**
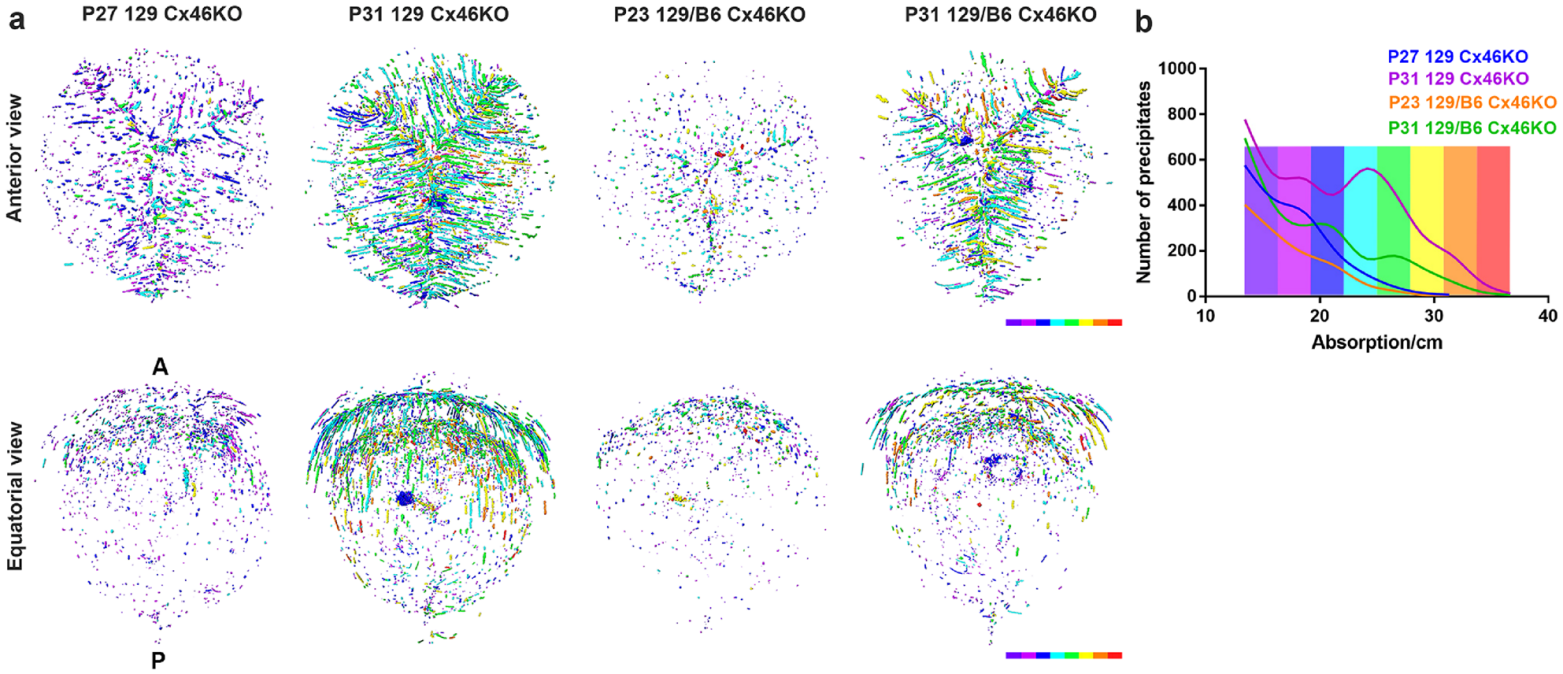
Absorption measurement and quantification of precipitates in Cx46KO lenses. The same individual Cx46KO lenses in Fig. 2 were analyzed. (**a**) Precipitates were measured by absorption per centimeter value in the cataract regions of 129 Cx46KO (P27 and P31) and 129/B6 Cx46KO (P23 and P31) lenses. Individual precipitates, in both anterior and equatorial views, were colored based on the color bar, red/orange for high density precipitates and purple/blue for low density precipitates. Lens anterior (A) and posterior (P) poles were indicated on the left equatorial image. (**b**) A graph shows precipitate numbers in different absorption values, the color bars are the same as in a, representing the regions of all images in (**a**).

We further carried out absorption measurement and quantification of precipitates in these Cx46KO lenses (Fig. 4). Absorption per centimeter value is an indicator of the precipitate density. P27 129 Cx46KO and P23 129 Cx46KO had low-absorption precipitates enriched in anterior parts of the lens cores, while P31 129 Cx46KO and P31 129/B6 Cx46KO showed a dramatic increase of high absorption and stripe-shaped precipitates which were concentrated in the anterior Y-suture with two shell-like regions (Fig. 4a).

The number of precipitates was further examined for their absorption per centimeter values in a graph, which was arbitrarily divided into eight color bars—red represents the highest absorption while purple represents the lowest absorption (Fig. 4b). The majority of the precipitates in P23 129/B6 Cx46KO and P27 129 Cx46KO lens cores were below 20 absorption/cm, and no precipitates were over 30 absorption/cm. In contrast, a large portion of precipitates in P31 129/B6 Cx46 and P31 129 Cx46KO were over 20 absorption/cm and some precipitates were over 30 absorption/cm. In addition, P31 129 Cx46KO showed the highest absorption density and number of precipitates, with a peak number of precipitates around 25 absorption/cm. Thus, the results suggest that the precipitates are correlated with age-related nuclear cataracts in Cx46KO lenses.

### Alizarin red positive calcium precipitates accumulated in Cx46KO nuclear cataracts

The high X-ray absorption of these precipitates was consistent with the expected ion absorption like calcium precipitates. Thus, wild-type, 129 Cx46KO and 129/B6 Cx46 KO lenses were stained with Alizarin red which specifically binds to calcium precipitates in different cells or tissues.

**Figure 5:**
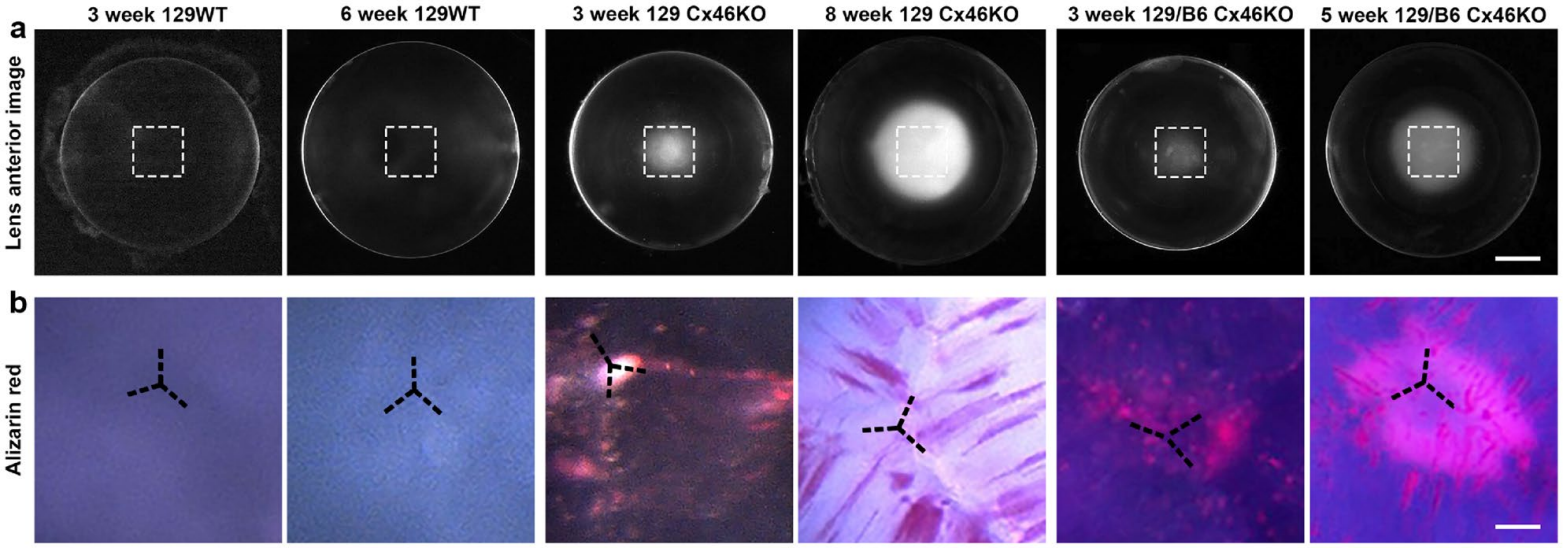
Images of different mouse lenses stained for calcium precipitates with Alizarin red. Three to five lenses from different mice of each genotype were used for staining. (**a**) Anterior view images of fresh 129 wild-type, 129 Cx46KO and 129/B6 Cx46KO lenses of mice at ages of 3–8 weeks under the dissecting microscope. Scale bar, 0.5 mm. (**b**) Anterior view images of Alizarin red stained lens nuclear regions, which were indicated by white dash boxes in (**a**). No Alizarin red staining was detected in 3-week-old or 6-week-old wild-type lens nuclear regions. Alizarin red staining revealed punctate signals in 3-week-old 129 Cx46KO and 129/B6 Cx46KO lens nuclear regions while uneven strip signals were mainly observed in 8-week-old 129 Cx46KO and 5-week-old 129/B6 Cx46KO lens nuclear regions. Y-shape dash lines indicate the lens sutures. Scale bar, 0.1 mm.

Alizarin red stained images of 129 WT, 129 Cx46KO and 129/B6 Cx46KO lenses from mice at 3–8 weeks old are shown (Fig. 5). Images of the same fresh lenses before the Alizarin red staining are displayed (Fig. 5a). Due to the penetration issue of Alizarin red staining solution, the lens cores were dissected out from these lenses for Alizarin red staining. The 129WT control lenses had no Alizarin red staining signals while all Cx46KO lenses displayed Alizarin red positive staining signals (Fig. 5b). Alizarin red stained signals appeared to be punctate dots in 3-week-old 129 Cx46KO and 129/B6 Cx46KO lenses. However, Alizarin red stained signals became uneven strips, which were observed at the anterior Y-suture regions in 5–8 weeks old 129 or 129/B6 Cx46KO lenses. Thus, the Alizarin red staining results further indicate that the accumulation of calcium precipitates occurs as an age-related event in both 129 and 129/B6 Cx46KO lens cores.

## Discussion

### X-ray tomographic imaging and cataract diagnosis

X-ray tomographic images reveal that micron-graded electron-dense precipitates appear only in Cx46KO lens cores, and the distribution and amount of the precipitates are correlated to age-related nuclear cataract formation. Alizarin red staining results further confirm the calcium precipitates in all Cx46KO lenses, which is likely an age-related event in Cx46KO lens cores. This study indicates that micron-sized calcium precipitates may directly cause abnormal light scattering in the Cx46KO lens cores. Thus, a loss of Cx46 leads to age-related calcium precipitation; this is a part of the novel mechanism to cause age-related nuclear cataract in Cx46KO mice.

Currently, slit-lamp microscopy is widely used in the clinical diagnosis of cataract^1^. Machine learning has been gradually applied in slit-lamp image processing and cataract classification^28,29^. Other technologies such as B-mode ultrasound, optical coherence tomography (OCT), and magnetic resonance imaging (MRI) are also used for lens examination^30,31^. MRI shows the size and some characteristics of cataracts encountered in pediatric and young adult patients^32^. OCT enables cross-sectional and three-dimensional visualization of ocular structures in the anterior segment and the retina^33,34^. However, these imaging technologies are restricted by resolution and unknown cause of light scattering.

The use of X-rays is gradually increasing in eye or lens imaging research. X-ray tomographic analysis can provide a submicron resolution 3D image of dense precipitates in cataracts of live lenses. A previous imaging study of the synchrotron X-ray photon in canine cataracts reported that X-ray imaging could efficiently characterize distributions of tiny light scattering calcification spots and extended areas of fiber cell compaction in the lens^35^. The dosage of X-ray in imaging needs to be used carefully to prevent the worsening of lens opacification^36^. A feasible study for high-resolution X-ray CT phase-contrast imaging on models of enucleated and intracranial rabbit eyes without injection of contrast agents was reported recently^22^. The unique characteristics of synchrotron radiation allow new advances in biomedical phase contrast imaging^19^. High-resolution 3D X-ray CT imaging of the internal structures of formalin-fixed primate eyes was compared with histological data^23^. All these studies indicate that high resolution X-ray tomographic imaging technology has great potential in clinical diagnosis of calcification related ocular pathology.

### Calcium homeostasis and calcium precipitates in connexin mutants

The relationship between calcium regulation and gap junction has been extensively studied, and the loss of Cx46 or Cx50 leads to a significant increase of lens calcium levels^14,37^. The distribution of calcium precipitates in X-ray tomographic images of Cx46KO lenses supports that nuclear cataract formation is partly associated with calcium mineralization. Recent studies have reported that Alizarin red-stained calcium precipitates are correlated with the morphology of nuclear opacity in mouse Cx46fs380 homozygous mutant lenses^38^; the Cx50D47A mutant lenses show a similar distribution in the lens core with enriched calcium precipitates in the anterior Y-suture regions^39^. Connexin mutations disrupt calcium homeostasis and cause biomineralization in cataract formation^40,41^. This work indicates that age-related progression of Cx46KO nuclear cataracts is correlated with the age-related increase of calcium precipitates in Cx46KO lens cores, especially in the anterior Y-suture regions. Calcium precipitates are enriched in anterior Y-suture regions of Cx46KO lens cores regardless of 129 and 129/B6 strain backgrounds.

Calcium precipitates are observed in various tissues and organisms in human diseases; studies have been conducted on calcium precipitates in human cataracts^42–46^. Calcium precipitates are also observed in animal models like canine cataracts^35^. Calcium is known to play essential roles in the development and physiology of the lens, and calcium elevation is involved in cataract formation^47^. It has been reported that ubiquitin K6 mutation alters unfolding protein response and perturbs gap junction function, resulting in calcium elevation, which induces calpain activation and cataracts^48^. Thus, calcium precipitation seems to be one of the general pathological events in different cataractous lenses.

### Molecular composition of calcium precipitates and lens opacity in Cx46 KO cataract

The molecular composition of calcium precipitates in Cx46KO lenses and in cataracts caused by other connexin mutations is unclear. Cataract formation associated with calcium precipitation may be one of the common pathological events in the lens during aging. Previous studies have reported that human cataractous lenses contain increased calcium oxalate or calcium carbonate crystals^45,49^. Calcium induced protein aggregation in human lenses has also been reported^50^. Based on the size, absorption density, and distribution of the precipitates in anterior suture, detected by X-ray tomography, these precipitates are likely to have directly and partly contributed to the light scattering of nuclear cataracts in Cx46KO lenses.

It is intriguing to observe two shell-like calcium precipitate layers in the anterior Y-suture regions. The lens diameter increase with age^3^, two calcium precipitate shell-like layers are estimated to be formed around neonatal stages. Thus, it is possible that fiber cells at the anterior suture region play an important role in the regulation of lens calcium homeostasis during postnatal lens development. It will be interesting to investigate whether the rapid growth of neonatal lenses needs a distinct regulatory mechanism of calcium homeostasis in the lens.

It has been suggested that the calcium elevation in Cx46KO lenses activates calpain, a calcium-dependent protease which cleaves crystallins. Cleaved crystallins lead to large abnormal protein aggregates that cause light scattering. It is unclear if calcium precipitates might be a secondary consequence of crystallin precipitation. Additional studies are required to address whether calcium precipitates and crystallin aggregates interact with each other during cataract formation in Cx46KO lenses. A recent study suggests that the imbalance of lens proteins causes lens opacity^51^. Major causes of light scattering in dense nuclear cataracts may be associated with crystallin degradation mediated by calcium activated calpains and lens protein imbalance, while calcium precipitates are likely contributing to some light scattering in restricted areas with accumulated calcium precipitates.

## Methods

### Cx46 knockout mice and nuclear cataracts

Mouse care and breeding were performed according to the Animal Welfare Regulations and the National Institute Health guidelines and regulations for using animal research and the ARVO Statement for the Use of Animals in Ophthalmic and Vision Research. The Animal Care and Use Committee (ACUC) at University of California at Berkeley approved the experimental protocol with all procedures as well as animal care, monitoring, and breeding for using mice in this study. This study is reported in accordance with ARRIVE guidelines for animal research as well. Three mouse lines, including 129SvJae strain (129) wild-type, and Cx46 knockout (Gja3^tm1^) at 129 SvJae strain background and at mixed background of 129Svjae and C57BL/6J strains^15,17^, were used in this project. Mice were housed with free access to food and water, with a 12:12 h light:dark cycle. Mice were euthanized by CO_2_ inhalation followed by cervical dislocation. Fresh lenses were immediately dissected in phosphate-buffered saline (PBS, 137 mM NaCl, 2.7 mM KCl, 10 mM Na_2_HPO_4_, 1.8 mM KH_2_PO_4_, pH 7.4) at room temperature and imaged at dark field background under a dissection microscope (Leica MZ 16, Germany) with a Zeiss AxioCam digital camera. The fresh lenses were immersed in PBS solution before the X-ray scanning analysis.

### X-ray phase-contrast imaging, image acquisition and reconstruction

The phase-contrast technique applied in this study was the propagation-based imaging technique (PBI), which requires a highly coherent and quasi-monochromatic X-ray beam available only at synchrotron radiation facilities. Micro-CT scanning was performed using a multilayer mirror monochromator generated monochromatic X-rays with energy of 20 keV from the synchrotron hard X-ray at beamline 8.3.2 of the Advanced Light Source, Lawrence Berkeley National Lab (Berkeley, CA). The X-ray wavefront transmits through a sample, generating a phase contrast image which can be recorded by a detector placed at a suitable distance from the sample. The intensity variations highlight the details embedded in the sample itself, thus enhancing their visibility^23^.

The procedure for μCT imaging of a fresh lens: before the scan, a Kapton tube with a diameter of 3 mm was cut into 2 mm length, and one end was blocked by hot-gluing a short metal rod. The fresh lens, dissected from enucleated mouse eyeball, was gently transferred into the tube filled with PBS solution using a pipette tip with a tweezer for adjusting the lens orientation. The tube was then mounted on a rotational stage in the μCT imaging hutch with the sample centered in the field of view. The scanning was conducted at room temperature. During the scanning, lens samples were in fixed positions without movement to ensure the image stack’s proper alignment. The lens sample was rotated at 0.125° incremental steps for a total of 180°. During the scan, the transmitted X-ray beam was recorded using a 50 μm thick LuAG:Ce scintillator to convert the X-rays into visible light, then imaged with an Olympus objective lens (10× or 4×), and a PCO.edge sCMOS detector. Each raw projection represents a two-dimensional X-ray attenuation map, which was used to reconstruct a 3D data volume. Image stacks (TIFF format) were produced using Xi-CAM^52^, with the gridrec algorithm as implemented in TomoPy^53^.

### X-ray tomographic visualization and statistical analysis

Using Avizo software from FEI (Waltham, MA), image stacks were 3D visualized, and 3D-reconstructions can be rotated and digitally sliced. Precipitate analysis was performed using the Dragonfly ORS software (Montreal, Canada). The image datasets were imported, and the imaging spacing (in μm) was set accordingly. Regions of interest (ROIs) were defined using a combination of algorithmic and manual methods, and multiple ROIs were created for statistical analyses. The parameters of individual precipitates were calculated by measurement of segmented voxels. The center point of precipitation was defined, and the distance to the center point of every precipitate was mapped for further color-coding. Individual precipitates in lens cataracts were color-coded and analyzed based on their parameters, such as Max Feret diameter, volume, aspect ratio, and absorption/centimeter. Images and parameter sheets were captured and exported from Avizo and Dragonfly. For statistical analysis, parameter datasheets acquired from Avizo and Dragonfly were imported to GraphPad Prism software (San Diego, CA), and visualized as scatter diagrams and line charts for analysis. The heatmaps were generated by a self-developed Python script in beamline 8.3.2. The statistical parameters used in this study were indicated in the results section.

### Lens whole-mount Alizarin red staining

A modified protocol based on previously published studies^38,54^ was used to perform whole-mount calcium staining of mouse lenses. Briefly, mouse lenses were fixed in 95% ethanol for 2 days with shaking at room temperature, followed by two overnight changes in 100% acetone. Lenses were treated with 1% (wt/vol) potassium hydroxide (KOH). Lens nuclear parts (about 60% of the lens size) were dissected out from treated lenses and were then incubated with freshly prepared 0.005% (wt/vol) Alizarin red with 0.05 M Trizma-base and 0.9% NaCl staining solution, pH 4. The staining reaction was closely monitored to avoid over-staining under a dissection scope. 1% KOH was applied to reduce background color for better visualization of stained calcium precipitation. Alizarin red stained lens samples were imaged under a Leica MZ16 dissecting scope with a digital camera.
